# Tetrodotoxin, a Candidate Drug for Nav1.1-Induced Mechanical Pain?

**DOI:** 10.3390/md16020072

**Published:** 2018-02-22

**Authors:** César Mattei

**Affiliations:** UMR CNRS 6015, INSERM U1083, Mitovasc Institute, Angers University, 49055 Angers, France; cesar.mattei@univ-angers.fr; Tel.: +33-2-44-68-82-74

**Keywords:** tetrodotoxin, Nav channels, Nav1.1, mechanical pain, nociceptor

## Abstract

Tetrodotoxin (TTX), the mode of action of which has been known since the 1960s, is widely used in pharmacology as a specific inhibitor of voltage-gated sodium channels (Nav channels). This toxin has contributed to the characterization of the allosteric model of the Nav channel, and to discriminating TTX-sensitive and TTX-resistant subtypes. In addition to its role as a pharmacological tool, TTX is now considered a therapeutic molecule, and its development should lead to its use in certain pathologies involving Nav channels, particularly in the field of pain. Specifically, the blockade of Nav channels expressed in nociceptive fibres is one strategy for alleviating pain and its deleterious consequences on health. Recent work has identified, in addition to the Nav1.7, 1.8 and 1.9 channels, the Nav1.1 subtype on dorsal root ganglion (DRG) neurons as a crucial player in mechanical and non-thermal pain. The sensitivity of Nav1.1 to TTX could be exploited at the therapeutic level, especially in chronic pain conditions.

## 1. Introduction

The mode of action of tetrodotoxin (TTX) was characterised in the early 1960s by the pioneering work of the Narahashi team, which made it clear that this non-peptidyl toxin inhibits voltage-gated Na^+^ channels (Nav) at very low concentrations [1]. Since then, the interaction between this toxin and the Nav channels has been dissected at the cellular and molecular level, and TTX is used as a pharmacological tool for Nav-dependent mechanisms [2]. In this respect, the blocking it exerts on these channels and its ability to inhibit the rise of the action potential of excitable cells are the cause of human intoxication after eating ‘fugu’ puffer fish. The second advance was the discrimination of TTX to certain subtypes of Nav channels. While blocking most Nav channels with high affinity, heart Nav1.5 and dorsal root ganglion (DRG) sensory fibres Nav1.8-1.9 subtypes show relative sensitivity or even resistance to TTX, respectively [3,4,5]. This discrimination that can be made between TTX-sensitive (Nav1.1, 1.2, 1.3, 1.4, 1.6, 1.7) and insensitive (Nav1.5, 1.8, 1.9) channels is undoubtedly a way of progress in the use of TTX as a therapeutic tool, particularly in the field of pain. It is indeed possible to specifically target the nociceptors, i.e., neurons responsible for converting high-intensity thermal, chemical or mechanical information into an electrical message. The transmission of this message is ensured by the propagation of the action potential, which depends on the opening of certain Nav channels. Blocking these channels located in nociceptors can relieve pain. In addition, TTX does not cross the blood brain barrier, or very poorly, reducing the probability of central system depression and any adverse effects [6].

## 2. Implication of Nav Channels in Nociception

**Figure 1:**
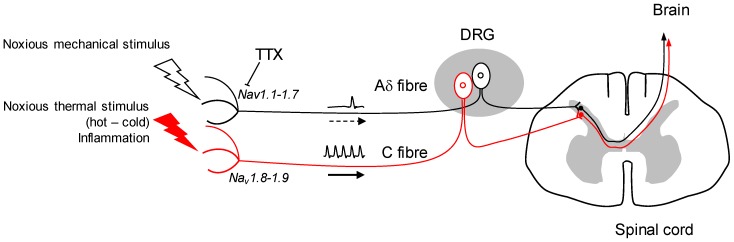
A simplified view of the putative use of TTX in the context of mechanical pain. Free nerve endings express receptors that can be targeted at high intensity by mechanical stimuli in acute forms of pain, or at low intensity in hypersensitivity-related pain disorders (mechanical hyperalgesia or allodynia). These mechanically activated Aδ fibres are supposed to express mainly Nav1.1 and Nav1.7 channels. They can be inhibited by TTX. Next are thermal-sensitive free terminals of C fibres, which are active during stimulations at high or low temperatures, and during the inflammatory process. They are TTX-insensitive. The therapeutic benefit of local administration of TTX treatment would come from the inhibition of mechanically activated nociceptors to relieve associated pain disorders.

The Nav channels of excitable cells allow the propagation of the action potential, and therefore the neuronal communication from the peripheral nerve terminal to the nerve centres (Figure 1). The nine subtypes of Nav channels are differentially expressed in the central and peripheral nervous system [7]. In terms of sensory fibres (DRG), there are mainly three of them present: Nav1.7, Nav1.8 and Nav1.9 [8]. They have long been proven to be therapeutic targets for pain. Many studies have demonstrated the coupling between these subtypes of Nav channels and nociceptors, in relation with major forms of pain, as well as heritable pain disorders [9,10,11]. Nav1.7 is a pivotal player in acute and inflammatory pain with good sensitivity to TTX (IC50 2 nM) [9]. The Nav1.8 and 1.9 TTX-resistant channels are predominant in nociceptive unmyelinated C fibres and are associated with the perception of nociceptive cold and pain at cold temperatures [10], and hypersensitivity to inflammatory pain [11], respectively. 

The Nav1.1 channel was, until recently, not considered an important player in the mechanisms of pain [12]. It is mainly present in the CNS, and the channelopathies related to a loss-of-function of Nav1.1 are relative to the autistic spectrum, migraines, or aura, notably the Dravet syndrome [13,14]. Conversely, a mutation of this channel with gain-of-function leads to a familial form of hemiplegic migraine [15]. However, the Nav1.1 channel, in addition to being present in the CNS, is expressed in the DRG sensory A fibres [16]. Recently, it has been shown that it is expressed in myelinated Aδ fibres [17], the role in mechanical nociception of which is―among other things―as high-threshold mechanical nociceptors [18,19]. In addition, a large majority of Nav1.1-positive fibres also express Nav1.7, these two isoforms being sensitive to TTX [17]. Finally, Nav1.1 plays a central role in the mechanical hypersensitivity observed in irritable bowel syndrome, experimentally-induced in mice. Nav1.1 therefore appears to be an important player in acute pain and mechanical allodynia, and not involved in inflammatory pain. Additionally, Nav1.1 expression is up-regulated in DRG after peripheral nerve injury, suggesting its involvement in the development of neuropathic pain [20]. Until now, it was well established that Nav1.8-1.9 isoforms are down-regulated, and Nav1.3 is up-regulated in neuropathic pain, but the Nav1.1 expression itself was not characterised.

## 3. Therapeutic Use of TTX

Other studies will confirm Nav1.1’s role in mechanical nociception or other forms of acute and chronic pain, but it appears to be a potential therapeutic target. The TTX targeting of mechanical pain by the inhibition of Nav1.1 in positive A fibres, while leaving the functional integrity of Nav1.8-1.9-expressing C fibres, seems a potential strategy in the future (Figure 1). Further research will determine whether Nav1.1 is up-regulated in chronic forms of mechanical pain, and if so, TTX could be a useful tool to alleviate such conditions. The use of TTX as an analgesic drug in neuropathic and inflammatory pain shows undeniable benefits in animal model studies [21]. It has also been tested by intramuscular injection to reduce cancer-related pain with significant effects [22,23]. Moreover, it has been challenged for its potential against drug addiction behaviours: TTX exhibits a reduction of cue-induced increases in heroin craving and drug-associated anxiety with no sign of cardiovascular side effects [24].

Until now, this use of TTX was based on its ability to block Nav channels, the expression of which is modified over time, implying these Nav channels―i.e., Nav 1.3, 1.7―to be therapeutic targets for chronic pain. In fact, TTX is in phase III clinical development for the management of cancer-related and neuropathic pain. Several preclinical and clinical studies have reported its ability to reduce pain conditions, although data may be a matter for debate [21]. Studies in humans show a benefit of TTX by local intramuscular or subcutaneous injection in the management of cancer pain [22,25]. A recent study has shown that a subcutaneous injection of TTX significantly reduces the reversal of localised mechanical hypersensitivity by capsaicin in a visceral mouse pain model, without implication of Nav1.7 [26], confirming data generated on Wistar rats, where TTX reduces mechanical allodynia in somatic neuropathic pain tests [27]. The management of pain with TTX might be considered in the future for its capacity as not only blocking several Nav channels, but also to target up-regulated Nav in pathological conditions. 

## 4. Conclusions

Several elements support the therapeutic use of TTX: on the one hand, when someone experiences pain (tendonitis, osteoarthritis, neuropathic pain), especially when it is prolonged in time, as in the case of hyperalgesia/mechanical allodynia, he/she wants to be relieved because chronic pain generates a disability to normal existence, which makes pain a debilitating condition. Elimination of pain may therefore be accompanied by adverse effects that may locally affect non-nociceptive sensory fibres. Several clinical studies conducted in patients with chronic pain have shown a long-term benefit of subcutaneous or intramuscular injection of TTX, despite adverse effects that resolved rather quickly through the treatment [22,23]. The TTX-mediated reduction of visceral mechanical pain did not induce any motor deficiency/incoordination [26]. These data suggest that there is a clear benefit of TTX over pain, and few consequences on sensory functions. On the other hand, the therapeutic use of TTX is made possible because the conditions associated with certain chronic pain show precisely a differential expression of the TTX-sensitive and TTX-resistant Nav channels at the nociceptors. Notably, tissue inflammation induces overexpression of TTX-sensitive Nav1.3 and Nav1.7 channels in nociceptors [28,29]: blocking these channels by TTX would reduce pain without affecting the neighbouring sensory functions. As for Nav1.1, it has yet to be determined whether it is differentially expressed in the case of chronic mechanical pain states.

There is no consensus around the use of TTX as a pain killer, most likely because (i) there are many paradigms of pain, both experimental and human, (ii) the administration of TTX should be done to what might be called the “right dose”, that is, an effective concentration to eliminate the pain without eliminating other sensory modalities, (iii) the route of administration of TTX must allow it to be delivered to the site of propagation of the nociceptive message to stop it, (iv) the possible combination of TTX with other analgesic molecules, (v) and the choice of TTX analogs or metabolites to increase the efficiency of inhibition of targeted Nav channels. 

However, given the recent evidence in the field of pain disorders, and in view of the current development of TTX as a therapeutic, it can be assumed that the management of chronic mechanical pain, including hyperalgesia and allodynia mechanical troubles, as well as channelopathies related to overexpression or a gain-of-function of TTX-sensitive Nav channels, could have a favourable outcome.
